# Workplace Psychiatric Disorders: Analysis of Socio-Professional Risk Factors and Fitness Outcomes among Tunisian workers

**DOI:** 10.1192/j.eurpsy.2025.1804

**Published:** 2025-08-26

**Authors:** G. Ben Rejeb, N. Kammoun, S. Ernez, S. Haj Ali, S. Fehri

**Affiliations:** 1 Tunisian Occcupational Safety and Health Institute; 2 University Tunis El Manar, Faculty of medicine of Tunis, Tunis, Tunisia

## Abstract

**Introduction:**

Psychiatric disorders in the workplace represent a major challenge for occupational medicine, affecting workers’ health and their professional capacity.

**Objectives:**

The study aims to analyze the socio-professional characteristics, risk factors, clinical evolution, and work fitness of patients with psychiatric disorders in the workplace.

**Methods:**

A retrospective analytical study was conducted on 62 medical files from the Institute of Health and Safety at Work (ISST) in Tunisia, covering the period 2023-2024. Sociodemographic, professional, medical data, and fitness decisions were extracted. Descriptive statistical analyses were performed to identify significant correlations.

**Results:**

The study included 62 cases. The average age was 44 years. The sex ratio was 0.8.

The most represented sectors were industry (29%), healthcare (24%), and education (11%). Depressive syndrome was the most frequent diagnosis (55%), followed by anxiety disorders (29%) and bipolar disorder (10%). Psychosis was found in 6% of cases.

A significant correlation between the industrial sector and the prevalence of depressive syndrome was found (p<0.01).

The predominant occupational risk factors included stress (51.6%), shift work (22.6%), and exposure to chemicals (16.1%). A positive correlation was observed between exposure to chemicals and the onset of anxiety disorders (p=0.03).

Analysis of fitness decisions showed that 41.9% of cases were deemed fit with accommodations, 29% temporarily unfit, 19.4% fit without restriction, and 9.7% permanently unfit.

**Conclusions:**

This study highlights the high prevalence of psychiatric disorders in the workplace and their significant impact on work fitness. The results underscore the importance of implementing targeted prevention strategies to preserve workers’ mental health and maintain their professional fitness.

**Disclosure of Interest:**

None Declared

